# A novel molecular imprinting polymer for the selective adsorption of D-arabinitol from spiked urine

**DOI:** 10.3906/kim-2002-56

**Published:** 2020-10-26

**Authors:** Yuni RETNANINGTYAS, Ganden SUPRIYANTO, Ni NYOMAN TRI PUSPANINGSIH, Roedi IRAWAN, Siswandono SISWODIHARDJO

**Affiliations:** 1 Faculty of Pharmacy, University of Jember, Jember Indonesia; 2 Department of Chemistry, Faculty of Science and Technology, University of Airlangga, Surabaya Indonesia; 3 Department of Pediatrics, Faculty of Medicine, University of Airlangga, Surabaya Indonesia; 4 Department of Pharmaceutical Chemistry, Facultyof Pharmacy, University of Airlangga, Surabaya Indonesia

**Keywords:** MIPs, D-arabinitol, bulk polymerization, noncovalent, selective adsorption

## Abstract

In this research, molecular imprinting polymers (MIPs) for D-arabinitol were synthesized using a bulk polymerization method through a noncovalent approach. The MIPs were prepared by using D-arabinitol as a template, acrylamide as a functional monomer, ethylene glycol dimethacrylateas cross-linker, benzoyl peroxide as an initiator and dimethyl sulfoxideas a porogen. MIPS was synthesized in several formulas with a different molar ratio of template to functional monomers and cross-linker. Fourier-transform infrared spectroscopy (FT-IR) and scanning electron microscopy (SEM) were used to characterize the MIPs produced. A batch rebinding assay was used to test the binding efficiency of each formula. Batch rebinding test results revealed that MIPsF3 with a molar ratio of the template: monomer and crosslinker ratio respectively (1: 4: 25) had the highest binding capacity at 1.56 mgg ^-1^ . The results of isotherm adsorption showed that the MIPs produced followed the Freundlich equation with an R-value of 0.97. The MIPs produced was also selective toward its isomeric compounds (i.e. L-arabinitol, adonitol, xylitol, and glucose). The extraction efficiency of the MIPs against D-arabinitol was 88.98%.

## 1. Introduction

D-arabinitol is a typical metabolite product of several *Candida* species that are pathogenic. The levels of D- arabinitol in serum and urine increase if the fungus *Candida* proliferates in the organism and causes invasive candidiasis [1]. In the late 1970s, high levels of D-arabinitol were found in the blood of systemic candidiasis patients. This fact made D-arabinitol potentially useful as a diagnostic tool in candidiasis patients. However, the incorporation of D-arabinitol as a diagnostic tool ultimately achieved a low specificity because the increased levels of D-arabinitol in the blood were not only found in candidiasis patients but also patients suffering from kidney dysfunction. To avoid false-positive results due to kidney damage, D-arabinitol levels are usually expressed in terms of the ratio D-arabinitol to creatinine or the ratio of D- to L-arabinitol [2–9].

Determination of the creatinine to the D-arabinitol ratio and D- to L-arabinitol ratio requires a separation method that can effectively separate D- and L-arabinitol because both are present in urine in the form of enantiomers.D-arabinitol levels in body fluids are quite low. The normal level of D-arabinitol in serum is around 0.2 μg mL-1 while in urine it is around 10 μg mL-1 [1], so to make D-arabinitol a diagnostic tool requires a sensitive and selective method. Early studies used gas chromatography (GC) or GC-mass spectrometry to detect and quantify D-arabinitol in serum [10–13]. However, these methods require expensive equipment, and specimen processing and analysis require considerable time and effort. To remediate this deficiency, several fluorometric or spectrophotometric enzyme assays using recombinant protein D-arabitol dehydrogenase were developed [14–16,3]. The main draw back of these analytical procedures is the unstable and tedious application in the preparation of biological recognition materials. Another method involves the electrochemical determination of D-arabitol [17]. Unfortunately, selectivity, especially to stereoisomers, of this chemosensor is low. Recently, an electric chemosensor using QCR and EG-FET based on polymers molecularly imprinted through the covalent approach has been successfully developed. Both chemosensors reveal significant enantio- and stereoselectivity with respect to D-arabinitol. Nevertheless, this method has a disadvantage related to the limits of detection of this method. It is higher than the level of the D-arabitol concentration in the urine of patients infected with candidiasis [18]. Therefore, there is a clear need to develop a suitable, selective, and sensitive determination of D-arabinitol in real samples. One method of separation that is quite effective for separating enantiomeric compounds involves using molecularly imprinted polymers (MIPs), even when the compound is in a complex matrix, as in amino acid enantiomers [19]. For that, in this research, we developed molecularly imprinted polymers as a separate joint sample with liquid chromatography-mass spectroscopy (LC-MS).

MIPs are synthetic polymers having specific cavities planned for a template molecule, which are synthe- sized by the copolymerization of functional and cross-linking monomers in the presence of a target analyte or template. In the most common preparation procedure, monomers make a complex with a template in the course of covalent or noncovalent interaction [20]. In this research noncovalent imprinting or self assembly approach is adopted during polymerization. In a noncovalent printing system, a mixture of prepolymerization consisting of templates and functional monomers will occur through noncovalent interactions. Noncovalent printing is much more flexible in terms of binding sides and can be diplomatic in a variety of models. Besides, the noncovalent approach is experimentally more straight forward relative to the covalent printing method because the stage of its complexity is achieved only by mixing the template with functional monomers in the appropriate solvent. Such did not require template derivatization, and template removal is usually sufficient by washing polymers [21]. One of the most reliable modern analytical methods is liquid chromatography tandem-mass spectrometry (LC-MS), which has advantages of both accurate identification and quantification of analyte target.

Herein we present a simple and straight forward method for the performance detection of D-arabinitol based MIPs as selective sorbents for extraction and further determination of D-arabinitol from urine by LC-MS as sensitive and accurate identification and quantification of analyte target. This work aims to synthesize a selective MIPs, a noncovalent approach was adopted to bind D-arabinitol in MIPs reversibly for its application to the treatment of complex matrices. This scheme as MIPs permits the sensitive, uncomplicated, and inexpensive separation and determination of D-arabinitol in urine samples by LC-MS.

## 2. Materials and methods

### 2.1. Materials

All chemicals were ofanalyticalgrade, D-arabinitol, acrylamide,ethylene glycol dimethacrylate (EGDMA), and dimethyl sulfoxide, L-arabinitol, xylitol, glucose, and adonitol were purchased from Sigma-Aldrich Co. Ltd. (St. Louis, MO, United States); while benzoyl peroxide, methanol (MeOH), and chloroform were purchased from Merck (Kenilworth, NJ, USA); and acetic acid was purchased from Alpha Chemika (Mumbai, India).

### 2.2. Equipment

TheBranson 2510 ultrasonic cleaner from Marshall Scientific (New Hampshire, USA) was used to disperse the mixtures.Fourier-transform infrared spectroscopy (FT-IR) spectra of polymer particles were recorded with Bruker Alpha ATR eco Ge from Bruker (Billerica, MA, USA). EBA20-Hettich was used to centrifuge and separate the polymer particles from the solution. Scanning electronmicroscopeInspect S-50; FEI Company, (Hillsboro, OR, USA) was used to study the morphology of polymer particles. Shimadzu LC-MS 2020 from Shimadzu (Tokyo, Japan) for reversed-phase high-performance liquid chromatography (LC-MS) on a SunFire C _18_ with 5 μm particle size. 4.6 mm internal diameter and 250 mm length (Waters, Milford, USA) column were used in the quantitation of D-arabinitol.

### 2.3. Synthesis of MIPs and NIPs of D-arabinitol

The synthesis of MIPs D-arabinitol was completed by the following procedure: 1 mmol of a template (D- arabinitol), 4 mmol of monomers (acrylamide), 20 mmol of cross-linker (EGDMA), and 50 mL of porogen (DMSO) were inserted into 150 mL Erlenmeyer, then stirred until a homogeneous solution was formed. Next, the initiator, 1% b/v benzoyl peroxide in chloroform was added and stirred until homogeneous. This solution was purged with nitrogen gas for 15 min to remove air bubbles so that the dissolution process could run perfectly. Then, the Erlenmeyer was closed and placed in a water bath. Polymer synthesis was carried out at 60 ◦ C for 12 h.

Nonimprinted polymers (NIPs) were synthesized with the same procedures and conditions as those used in MIPs synthesis but without the addition of a template. The same procedures and conditions were also incorporated into the synthesis of other imprinted polymer D-arabinitol polymers with varying compositions of acrylamide and EGDMA using the free radical solution polymerization method, referred to as MIPs F_1_, MIPs F_2_, MIPs F_3_, and MIPs F_4_. Variations in the form of D-arabinitol MIPs synthesized in this study are shown in Table 1. The polymers formed (MIPs and -NIPs) were then filtered out with a vacuum pump and oven at 40 ◦ C until dry and crushed to achieve a particle diameter of 50 mm or smaller.

**Table 1:** The composition of MIPs and NIPs formulas in various variations.

Composition	MIPs F_1_	MIPs F_2_	MIPs F_3_	MIPs F_4_	NIPs
Template (mmol)	D-arabinitol (1)	D-arabinitol (1)	D-arabinitol (1)	D-arabinitol (1)	D-arabinitol (1)
Functional monomer (mmol)	Acrylamide (4)	Acrylamide (5)	Acrylamide (4)	Acrylamide (5)	Acrylamide (4)
Cross-linker (mmol)	EGDMA (20)	EGDMA (20)	EGDMA (25)	EGDMA (25)	EGDMA (20)
Porogen (mL)	DMSO (50)	DMSO (50)	DMSO (50)	DMSO (50)	DMSO (50)

The extraction methods referred to [22] with modification. D-arabinitol molecules were extracted by washing the MIPs with methanol:acetic acid (4:1, v/v) for five rounds and then with methanol until the template was not detected by LC-MS m/z 152.

### 2.4. Batch rebinding assay

The determination of the binding efficiency of polymers to D-arabinitol was done by Batch rebinding assay. MIPs from each formula and NIPs of 0.050 g were added to the vial containing 10 mL of 8 mgL^-1^ D-arabinitol solution in water [defined as initial D-arabinitol (Co)]. This sample was then incubated by stirring at room temperature. Each sample was incubated at different time intervals (1, 2, 3, 4, 5, 6, or 7 h) for each formula. After incubation, the sample was centrifuged at 5000 rpm for 10 min to separate the liquid and the solid forms. After being separated from the solid material, the concentration of the remaining substrate in the solution was then measured [defined as free D-arabinitol (Ci)]. The determination of this concentration was carried out using LC-MS. NIPs used as controls were defined as nonspecific bonds. The amount of D-arabinitol bound to MIPs was calculated by reducing the Co with Ci. The MIPs binding capacity of each formula and NIPs were calculated using the following equation:

(1)Binding capacity(Q)=V(C0-Ci)W

where V is the volume of D-arabinitol solution added, Co (mgL^-1^) is the early D-arabinitol concentration, Ci (mgL^-1^) is the free D-arabinitol concentration after incubation, and W (g) is the weight of the particles MIPs/NIPs used [23].

### 2.5. Isotherm adsorption study

The determination of the corresponding isotherm equation was based on the correlation coefficient (R2) pro- duced by the linearity equation. In this study, the isotherm adsorption was carried out with a simple linear regression test using the Freundlich equation, which is described in Equations 2 and 3 below.

(2)Q=AX1/n

(3)logQ=logA+1nlogX

where Q is the binding capacity (mgg^-1^), A and n are the adsorption coefficients, X is the concentration of the non-bound analyte (μm), andR^2^is the correlation coefficient of the regression equation [24].

### 2.6. Competitive binding test

The selectivity of MIPs was tested by comparing the binding capacity of MIPs to D-arabinitol relative to the MIPs binding capacity of compounds that had similar structures as that of D-arabinitol. Compounds with similar structures included in this selectivity test were L-arabinitol, xylitol, adenitol, and glucose. Here, 0.05 g of the most optimal MIPs formula( F3) and NIPs F3 polymers were added with a 10 mL solution of D-arabinitol, L-arabinitol, xylitol, adonitol, and glucose separately in a concentration of 8 mLg ^-1^ . The same procedure was also completed for NIPs. All vials were incubated by shaker at 100 rpm for six hours. After incubation, each sample was centrifuged at 5,000 rpm for 10 min. The amount bound for each template was analyzed by LC-MS. The distribution ratio (KD) of D-arabinitol (mLg^-1^) between MIPs or NIPs with solvents (water) was determined using the equation (4):

(4)K=(Ci-Cf)VCiW

where Ci (mgL^-1^) was the initial concentration of D-arabinitolin the solution Cf (mgL^-1^) was the final concentration of D-arabinitol in the solution, V (mL) was the volume of solvents. W (g) was the mass of MIPs or NIPs [25].

### 2.7. Chromatographic conditions

The LC-MS was conducted by using the C18 column (150 ×4,6 mm, 5 μm) with the mobile phase consisting of acetonitrile and distilled water in the ratio of 80:20 v/v, respectively. The flow rate was set at 0.5 mL/min with m/z detection at 152 and the injection volume was set at 20 μL.

### 2.8. Method validation of MIPs LC-MS

The method validation was performed for linearity, range, the limit ofdetection (LOD), limit of quantification (LOQ), accuracy, and precision. All the following data were obtained after the treatment of samples by using the optimized MIPs LC-MS condition. The calibration was established by preparing different concentrations of the standard solution (1–13 μgmL^-1^) before the MIPs procedure. LOD and LOQ, defined as the concentration corresponding to a signal equal to 3 and 10 times the standard deviation of the blank. Accuracy was evaluated using recovery assays carried out. The precision was evaluated by measuring relative standard deviations (RSD%).

### 2.9. Extraction of D-arabinitol from spiked human urine

Fresh urine collected from humans who did not take medication was centrifuged and filtered. Pipets 2 mL of the samples into 10 mL volumetric flask. One sample was spiked and the other was not. The spiked sample was combined with water and D-arabinitol in a 10 mL of the volumetric flask to obtain a solution with a D-arabinitol concentration of 8 mgL^-1^and the unspiked sample combine with water until 10 mL. The spiked sample and unspiked sample then were added to vials containing 0.050 g of MIPs F3 and incubated for 6 h . Next, each sample was centrifuged at 5000 rpm for 10 min to separate the solid and the liquid materials. The solid part was then washed with water and extracted using 10 mL of water:methanol (4:1 v/v) solution to release the D-arabinitol bound to MIPs. The extracted liquid was then measured to determine its D-arabinitol content using LC-MS to elucidate the D-arabinitol from MIPs [25]. The recovery is calculated by equation 5

(5)R(%)=Cspikemeasured-CunspikemeasuredC addedX100%

## 3. Results and discussion

**Figure 1:**
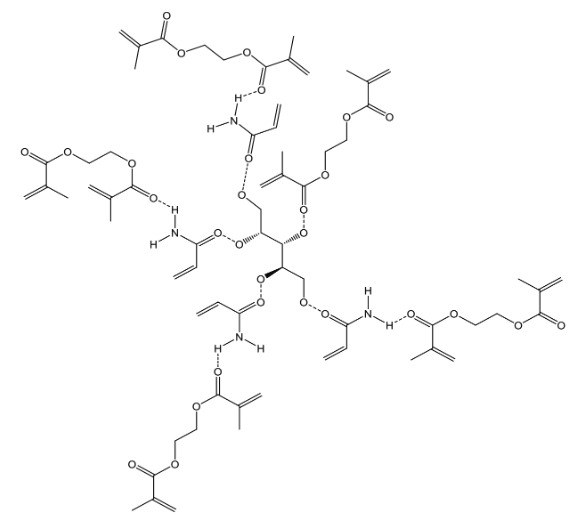
Illustrated of D-arabinitol MIPs synthesis results.

MIPs synthesis results in the form of a white hard polymer block with a brittle structure. Polymer synthesis was carried out by mixing D-arabinitol templates with acrylamide as functional monomers. The reaction of polymer formation between functional monomers and template occurred in situ in noncovalent interactions. This treatment was intended to elucidate the interaction of hydrogen bonds between D-arabinitol with acrylamide, where the interaction occurred at the O atomic ketone with the H nuclear hydroxy group at positions one and five of D-arabinitol. This stage was the prepolymerization stage. After the prepolymerization process occurred, EGDMA was added to this mixture, which functions as a cross-linker, so that the copolymerization process can begin. The initiator used in this MIPs synthesis was a benzoyl chloride solution in chloroform with DMSO acting as a porogen. MIPs were made in a variety of compositions from functional monomers and cross-linker. MIPs synthesis results were illustrated in Figure 1. NIPs were made in the same way but without the addition of template [25,23].

### 3.1. MIPs and NIPssynthesis results characterization

#### 3.1.1. FTIR characterization

**Figure 2:**
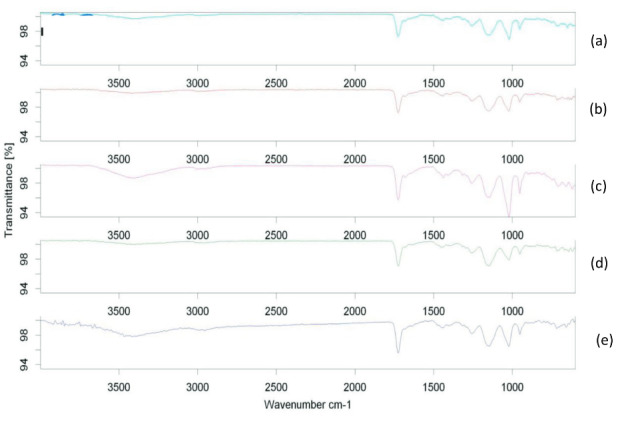
Spectra of FT-IR: MIPs F1 (a), MIPs F2 (b), MIPs F3 (c), MIPs F4 (d), and NIPs (e).

One of the characterizations of MIPs was done by analysis with FT-IR. FT-IR is an essential analytical method used to identify any groups contained in the MIPs or NIPs synthesis results. The FT-IR MIPs spectra of each formula and the NIPs are shown in Figure 2.

Based on Figure 2, there was a weak peak in the range of wave numbers 3399.72 to 3429.48 cm − 1, which indicated the presence of N-H vibrations from acrylamide, which was used as a monomer in the synthesis of MIPs and NIPs. The existence of peaks in the region of wave numbers 2953.20 to 2991.52 cm^-1^ indicated the presence of vibrations of C-H stretching from symmetrical and asymmetrical aliphatic compounds from MIPs and NIPs. The presence of a distinctive and sharp peak in wave numbers 1725.32 to 1725.85 cm^-1^ indicated the presence of vibrations of the C=O stretching carbonyl group of acrylamide on MIPs or NIPs. The presence of D-arabinitol templates in the MIPs F1, MIPs F2, MIPs F3, and MIPs F4 polymers produced characterized by a weak peak in the region of wavenumber 2955.75 cm^-1^ indicated the presence of C-H stretching vibrations from the template. The peaks in the wavenumber region of 1257.78 to 1258.96 cm^-1^ on MIPs and NIPs showed the presence of symmetrical stretching –O-CH 2 -O- vibrations. The vibrations in the 700 to 600 cm^-1^ wave number region indicated the presence of OH bending from D-arabinitol. The FTIR spectrum of MIPs showed that the MIPs produced were functional groups an alloy of amine, carbonyl, and hydroxyl and as expected. The FT-IR NIPsspectrum showed that, in the resulting polymer, there was not a D-arabinitol template because the spectra in the fingerprint region did not have absorption in the wavenumber region of 600 cm^-1^[26].

#### 3.1.2. Scanning electron microscopy (SEM) characterization

**Figure 3:**
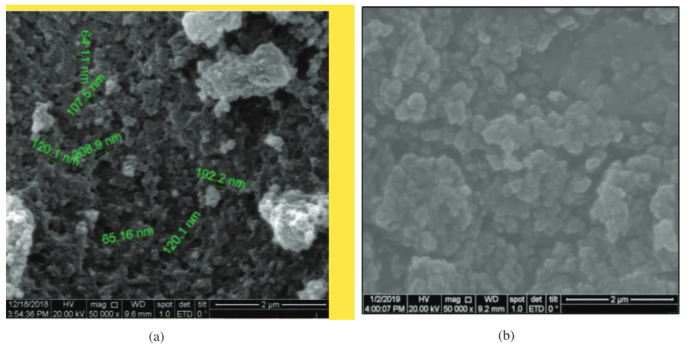
SEM of MIPs (A) and NIPs (B).

SEM analysis aimed to determine the surface morphology of the MIPs and NIPs synthesized [27]. Samples analyzed by SEM included MIPs F3 and, as a control or comparison, NIPs were used. The results of the analysis with SEM MIPs and NIPs at a magnification of 50000×are shown in Figure 3.

Based on Figure 3, SEM analysis shows morphological differences between MIPs and NIPs. MIPs had an irregular, rough morphological structure, while the NIPs had a regular morphological structure with a smooth surface. Irregular MIPs indicated that the polymer had cavities due to template removal, where the cavities served to recognize molecules with the same size, structure, and physicochemical properties as in D-arabinitol,while regular NIPs structure indicated that there were no specific binding sites formed by the template.

### 3.2. Batch rebinding assay

**Figure 4:**
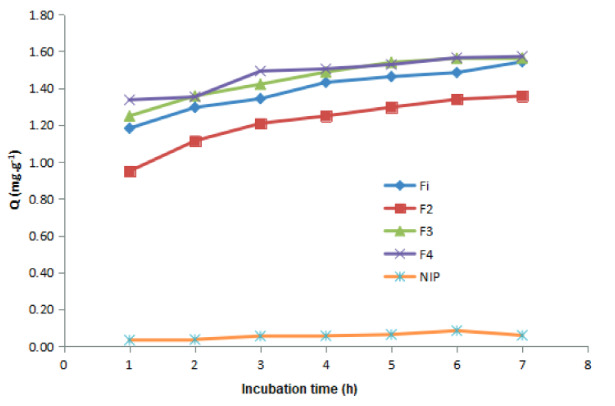
Batch rebinding test results for each MIPs (F1–F4) and NIPs formula.

This test sought to determine the efficiency of binding MIPs of each formula and NIPs to D-arabinitol. The binding capacity of MIPs to the template in this study was carried out at optimum conditions per the results of the optimization that had been done before and the results were measured by LC-MS. The results of the measurement of binding efficiency at different time intervals for each of the MIPs and NIPs formulas for D- arabinitol are shown in the graph in Figure 4.

Figure 4 shows that the formulas that had the highest binding capacity were F3 and F4; both had a binding capacity of 1.56 mgg ^-1^ . Whereas, if seen from the yield produced, MIPs F3 had more return relative than MIPs F4 at 18.444 g, while the return produced from MIPs F4 was 6.32 g. Apart from that, the efficiency of the material used in MIPs F3 had a more efficient composition where the number of functional monomers used was less than MIPs F4. The most optimal MIPs formula was the most optimal comparison of template which had functional monomers and cross-circumference (1:4:25) because such could increase the effectiveness of the cavity in recognizing template molecules as well as the stability of the polymer framework and the similarity of cavity structures with template molecules. The binding capacity value of NIPs was minimal relative to MIPs F1, MIPs F2, MIPs F3, and MIPs F4, and this showed that NIPs did not have a cavity able to recognize template molecules so it could not bind to the template molecules. Functional monomers and useful cross-linker molecules were capable of stabilizing the functional monomer complexes and polymer structures. The ratio of functional template–monomers and the ratio of functional–molecular cross-linking monomers were crucial factors to consider in the synthesis of MIPs. The useful template–monomer rate will affect the number of complementary binding sites so that it would affect the effectiveness of the cavity in recognizing the template molecule, while the cross-linked functional molecular monomer ratio will affect the stability of the polymer skeleton and the similarity of the cavity structure to the template molecule [28].

### 3.3. Isotherm adsorption study

**Figure 5:**
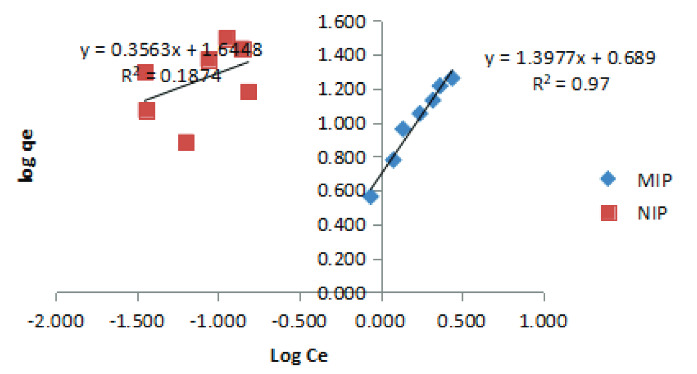
Graph of MIPs and NIPs adsorption isotherm with the Freundlich equation.

The adsorption isotherm could be used to study the effect of physicochemical parameters that occurred in an adsorption process and the adsorption mechanism. In this study, the isotherm adsorption study was carried out with a simple linear regression test using the Freundlich equation. Data from the calculation of the isotherm study of the Freundlich equation are shown in Table 2 and Figure 5.

**Table 2:** Isotherm adsorption parameter value for each equation.

Equation	Parameter	Adsorbent	
		MIPs	NIPs
Freundlich	n	0.715	2.807
	A (gkg^-1^)	4.887	44.137
	R2	0.970	0.187

Isotherm adsorption studies were carried out to understand the phenomenon of containment or mobility of an analyte from the solution to the solid phase at a constant pH and temperature [29]. The adsorption isotherm could be used to study the effect of physicochemical parameters that occurred in an adsorption process and the adsorption mechanism. In this study, the isotherm adsorption study was carried out with a simple linear regression test using the Freundlich equation. Data from the calculation of the isotherm study of the Freundlich equation are shown in Table 2 and Figure 5.

The resulting correlation coefficient showed that the adsorption isotherm of MIPs D-arabinitol followed the Freundlich isotherm equation with a correlation coefficient (R2) of 0.97, while the NIPs adsorption isotherm did not follow the Freundlich isotherm equation where the correlation coefficient (R2) was 0.1874. This Freundlich isotherm model showed that the adsorption that occurred was physisorption, meaning that the adsorbate was bound by the adsorbent physically with Van der Walls forces. Determination of the adsorption coefficient (A) could be roughly used as an indicator of adsorption capacity, while the value of 1/n represented the value of adsorption intensity. In general, it could be stated that the higher the value of A, the higher the value of the adsorption capacity, and, for the value of 1/n, smaller adsorption means better adsorption. The adsorption capacity value for MIPs D-arabinitol and NIPs were 4.88652 and 44.13671 gkg^-1^, respectively.

### 3.4. Selectivity test

The selectivity of MIPs and NIPs adsorbents was carried out using a batch system by conducting a series of tests with the addition of compounds typically the same as D-arabinitol with the same concentration in determining the absorption capacity of MIPs D-arabinitol and NIPs. The selectivity of MIPs D-arabinitol and NIPs to the test compounds was expressed as a distribution coefficient (KD).

**Figure 6:**
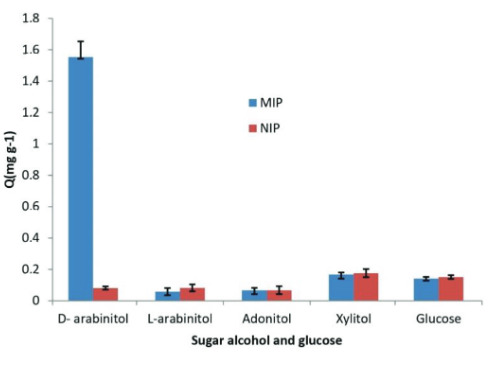
The adsorption capacity (Q) of MIPs and NIPs for each compound.

The KD -values of each compound for MIPs and NIPs are shown in Table 3 and the Q values of MIPs and NIPs for each compound are shown in Figure 6.

**Table 3:** KD value of each compound in MIPs and NIPs.

		
D-arabinitol	195.00	10.5
L-arabinitol	4.35	7.35
Xylitol	10.7	11.2
Adonitol	6.25	8.9
Glucose	19.075	20.5

Based on the data in Table3 and Figure 6,each compound had a different KD value, where the KD value of D-arabinitol to MIPs was much greater when compared to other compounds. This showed that the MIPs produced have a higher degree of similarity in chemical structure with the D-arabinitol than others analytes.

### 3.5. D-arabinitol assay in human urine samples

**Figure 7:**
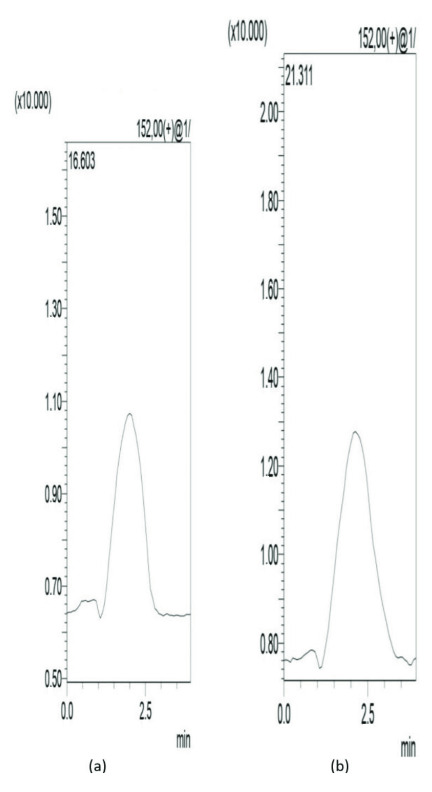
Chromatogram of D-arabinitol standard (a) and chromatograms obtained after clean up of an 8 μg mL 1 solution of D-arabinitol in urine samples with MIPs (b).

To demonstrate the applicability of MIPs for the selective clean-up of D-arabinitol, the MIPs was used to the purification of spiked human urine. Aqueous media was used for the loading solution and the washing protocol was estimated for obtaining maximum recovery of the analytes using water:methanol (4:1 v/v). The chromatograms obtained from urine samples are shown in Figure 7a. As shown in these figures, the retention time of D-arabinitol is about 1. 867 and the run time is 4 min and this well-organized method obtained cleaner extracts peaks from the complex biological matrices to be removed are shown in Figure 7b. Results from the LC-MS analyses demonstrated that the MIPs extraction of D-arabinitol for urine samples is linear in the ranges 1–13 μgmL ^-1^ . The linearity regression analysis way y=3140.8x+46017.3 with a correlation of 0.998. The limit of detection (LOD) and limit of quantification (LOQ) for D-arabinitol in urine samples was 0.25 and 0.83 mugmL^-1^ , respectively. The repeatability for 10 mL of spiked urine (8 μgmL^-1^ of D-arabinitol), expressed as RSD (n = 5), was lower than 4%. The recoveries for urine was 88.98 ±1.2% (n = 3).These results indicate that the method we developed here has an advantage when compared to the previous method [18] because it has a high selectivity to the enantiomer compounds of D-arabinitol and has high sensitivity so that it can be applied to the determination of D-arabinitol levels in urine samples.

## 4. Conclusion

MIPs, which were successfully synthesized in this research by the bulking polymerization method, showed good recognition ability of D-arabinitol in water media. MIPs F3 had the highest binding capacity of 1.56 mgg^-1^, −1 produced was also able to extract D-arabinitol contained in urine with a % recovery value of 88.98%. which was much more significant than NIPs, which only had a binding capacity of 0.08 mgg . The MIPs
